# STINGing the Tumor Microenvironment to Promote Therapeutic Tertiary Lymphoid Structure Development

**DOI:** 10.3389/fimmu.2021.690105

**Published:** 2021-05-13

**Authors:** Jessica N. Filderman, Mark Appleman, Manoj Chelvanambi, Jennifer L. Taylor, Walter J. Storkus

**Affiliations:** ^1^ Department of Immunology, University of Pittsburgh School of Medicine, Pittsburgh, PA, United States; ^2^ Department of Dermatology, University of Pittsburgh School of Medicine, Pittsburgh, PA, United States; ^3^ Department of Pathology, University of Pittsburgh School of Medicine, Pittsburgh, PA, United States; ^4^ Department of Bioengineering, University of Pittsburgh School of Medicine, Pittsburgh, PA, United States

**Keywords:** dendritic cells, immunotherapy, STING agonists, tertiary lymphoid structures, T cells, tumor, vaccine, vascular normalization

## Abstract

Tertiary lymphoid structures (TLS), also known as ectopic lymphoid structures (ELS) or tertiary lymphoid organs (TLO), represent a unique subset of lymphoid tissues noted for their architectural similarity to lymph nodes, but which conditionally form in peripheral tissues in a milieu of sustained inflammation. TLS serve as regional sites for induction and expansion of the host B and T cell repertoires *via* an operational paradigm involving mature dendritic cells (DC) and specialized endothelial cells (i.e. high endothelial venules; HEV) in a process directed by TLS-associated cytokines and chemokines. Recent clinical correlations have been reported for the presence of TLS within tumor biopsies with overall patient survival and responsiveness to interventional immunotherapy. Hence, therapeutic strategies to conditionally reinforce TLS formation within the tumor microenvironment (TME) *via* the targeting of DC, vascular endothelial cells (VEC) and local cytokine/chemokine profiles are actively being developed and tested in translational tumor models and early phase clinical trials. In this regard, a subset of agents that promote tumor vascular normalization (VN) have been observed to coordinately support the development of a pro-inflammatory TME, maturation of DC and VEC, local production of TLS-inducing cytokines and chemokines, and therapeutic TLS formation. This mini-review will focus on STING agonists, which were originally developed as anti-angiogenic agents, but which have recently been shown to be effective in promoting VN and TLS formation within the therapeutic TME. Future application of these drugs in combination immunotherapy approaches for greater therapeutic efficacy is further discussed.

## Chronic Inflammation and TLS Organogenesis: General Overview and Cancer Indications

TLS are non-encapsulated aggregates of lymphoid cells that form at sites of sustained inflammation, including tissues impacted by autoimmune disease, chronic infection, and cancer (1, 2). Recent findings suggest that the presence of TLS within tumor lesions positively correlates with favorable prognosis in most forms of solid cancer (1–4). TLS are associated with specialized vascular structures (i.e. HEV) that differentiate from CD31^+^ VEC or endothelial progenitor cells under pro-inflammatory, pro-angiogenic conditions (5–7). HEV express the cell surface marker peripheral node addressin (PNAd, a binding partner for CD62L expressed on lymphocytes) and produce chemokines (CCL19, CCL21, CXCL13), which facilitate the recruitment of naïve/central memory CD62L^+^CCR7^+^ T cells, naïve CD62L^+^CXCR5^+^ B cells, CXCR5^+^ T follicular helper (Tfh) cells and mature CCR7^+^ DC into the TME (1). In this context, it is believed that TLS serve as local sites for the *de novo (*cross)priming, expansion, and differentiation of tumor-specific T and B cells, leading to more efficient/effective anti-tumor responses within sites of active disease (2, 8–13). TLS also appear to define an operational site in which the T cell and B cell repertoires may expand their specificity against a broadened range of tumor antigenic targets, *via* the paradigms of epitope spreading or determinant spreading (14). Notably, TLS exhibit heterogeneity in their cellular composition and in the organization of their integrated cell subsets, which is believed to be reflective of their maturational status (2, 15). Classical (mature) TLS are characterized by the presence of i.) PNAd^+^ HEV surrounded by ii.) aggregates of T cells and mature DCs and iii.) distinct B cell zones containing naïve B cells around germinal center (GC)-like structures (1–3, 16, 17). Non-classical (immature) TLS contain some but not all of these three characteristics (i.e. typically lacking B cells/GC) (16). Strikingly, the presence of either classical TLS or non-classical TLS in TME portends superior prognoses in cancer patients (1–3, 10, 16–27).

## TLS Homeostatic Cytokines/Chemokines Are Modulated by Agents That Promote Vascular Normalization (VN) in Tumors

An important component of TLS formation in peripheral tissues is sustained local production of homeostatic chemokines that recruit immune cells into affected tissue sites and serve as cues for establishing organized interactions between infiltrating lymphocytes and antigen presenting cells (APC). This topic has been well-described in other publications (28, 29) and elsewhere in the current volume and is therefore only briefly discussed below.

One key homeostatic chemokine associated with TLS development is CXCL13 (also known as B lymphocyte chemoattractant [BLC] or B cell-attracting chemokine 1 [BCA-1]), the ligand for CXCR5 (28). The production of CXCL13 by tumor-associated fibroblasts, Tfh cells, follicular dendritic cells (FDC) and HEV is positively-correlated with the formation of GC that contain CXCR5^+^ B cells (29, 30). While not yet investigated in the tumor setting, forced expression of CXCL13 in normal pancreatic β cells leads to the formation of TLS containing HEV, B cells and T cells *via* a process dependent on the initial infiltration of B cells into tissue and the activation of the lymphotoxin (LT)αβ-LTβR signaling cascade (31). Two additional major TLS-associated homeostatic chemokines produced by mature DC and HEV are CCL19 and CCL21, both of which serve as ligands for CCR7 (28). In normal mouse pancreatic tissue, ectopic overexpression of CCL19 or CCL21 induces the formation of TLS containing CD4^+^ T cells, CD11c^+^ DCs, and B220^+^ B cells surrounding HEV *via* a process dependent on CCL19/21-induced expression of LTα_1_β_2_ complexes on CD4^+^ T cells (32). Although TLS were not formally evaluated in their endpoint analyses, several studies have shown that treatment of murine tumors by injection with recombinant CCL19, viruses encoding CCL19 or CCL21, or DC engineered to express CCL21 results in robust tumor infiltration by T cells and DC in association with slowed tumor growth and extended overall survival (33–36).

In addition to these chemokines, tumor necrosis factor (TNF), interferon (IFN) and interleukin (IL)-1 superfamily cytokines also play major roles in TLS neogenesis. Lymphotoxins (LTα/TNFSF1 and LTβ/TNFSF3) and LIGHT/TNFSF14 produced by immune cells play canonical roles in the formation of TLS (29, 37). Lymphotoxins form bioactive heterotrimers (LTα_3_, LTα_1_β_2_, LTα_2_β_1_) that bind to LTβR/TNFRSF3, with LTα_3_ also binding and mediating signals through the TNFR1/TNFRSF1A, TNFR2/TNFSF1B and herpesvirus entry mediator (HVEM)/TNFRSF14 receptors (38). LIGHT also binds to HVEM/TNFRSF14 (39). TNF receptors represent important signaling receptors for endothelial cell function and proliferation and they facilitate TLS neogenesis. TNFR1/2 expression on endothelial cells has been shown to be necessary for HEV formation and T cell infiltration into murine melanoma (40). Mice lacking TNFR1/2 on the endothelium or LTα on CD8^+^ T cells have significantly decreased PNAd expression, demonstrating that LTα_3_ engagement of TNFR1 induces PNAd expression on the tumor vascular endothelium (40). In kidney and pancreatic tissues, the forced overexpression of LTα promotes lymphoid aggregate formation (containing T cells, B cells, and APCs) and tumor vascular reprograming, as indicated by increased expression of VCAM-1, ICAM-1, MAdCAM, and PNAd on VEC/HEV (41). Additionally, combined overexpression of LTα and LTβ further enhances infiltration of naïve lymphocytes and expression of homeostatic chemokines when compared to LTα overexpression alone, suggesting the synergistic action of these cytokines in TLS formation (42). In line with such findings, B16.F10 melanoma-bearing mice treated with a tumor-targeted GD2 scFv-LTα fusion protein demonstrate increased densities of intratumoral HEV and develop a diverse T cell repertoire in association with TLS neogenesis (43). Remarkably, recent reports in murine transplantable and carcinogen-induced tumor models support the operational dominance of TNFR- over LTβR-mediated signaling for HEV/TLS neogenesis in the TME (40, 44), findings which contrast with the canonical importance of LTβR-mediated signaling for HEV/TLS formation in normal tissues and in ontogenic secondary lymphoid organogenesis (1, 29, 30, 40, 44). Beyond lymphotoxins, LIGHT activation of VEC has also been shown to play a role in TLS formation in cancer models. C57BL/6 mice bearing intracranial NSCG glioblastomas treated with a fusion protein encoding LIGHT and a vascular targeting peptide (LIGHT-VTP) displayed VN and induction of classical TLS within the TME (45). In murine fibrosarcoma models, forced expression of LIGHT prompted naïve T cell infiltration and local production of homeostatic chemokines, leading to tumor rejection in the therapy setting (8, 46).

Type I IFNs have also been reported to drive TLS formation in normal tissues (47, 48). In murine models, a subset of PDGFRα^+^ lung fibroblasts produce CXCL13 in response to infection with influenza virus or intranasal administration of IFNβ (48). IFN-I receptor (IFNAR) activation in these cells results in increased recruitment of CXCR5^+^ B cells and ectopic germinal center formation in the lungs, which in turn promotes the development of a broadly neutralizing repertoire of antiviral antibodies conferring cross-strain protection (48). In a hydrocarbon (TMPD)-induced model of autoimmune SLE, mice with intact IFN-I signaling had worse clinical scores and increased lupus-specific autoantibody production compared to IFNAR-deficient mice (49). It was shown that IFN-I produced by activated DCs in this model was associated with the formation of classical TLS containing B cells, CD4^+^ T cells, and DC along with coordinate expression of TLS homeostatic chemokines (CCL19, CCL21, CXCL13) and their receptors (CCR7, CXCR5) (50). Sustained IFN-I/IFNAR signaling in tissues has similarly been shown to promote TLS formation in additional studies *via* local production of pro-inflammatory CXCR3 ligand chemokines (CXCL10/11) and lymphotoxins (51, 52).

Furthermore, gene therapy delivering IL-1 family member IL-F9/IL-36γ induces HEV and TLS formation in mouse colon carcinomas in association with the development of superior T cell-mediated anti-tumor immunity and tumor growth suppression (11). Notably, the activation of IL-36R on immune and stromal cell populations has been shown to upregulate local production of pro-inflammatory, pro-TLS factors including CXCL10, LTα and IFNs (53). In humans, IL-36γ is expressed by the tumor vasculature in colorectal cancers and has been correlated with an increased density of CD20^+^ B cells localized in TLS in these tumors (54).

## STING Signaling Enforces a Pro-Inflammatory, Pro-TLS TME

STING (STimulator of INterferon Genes) is a cytosolic DNA sensing protein that is activated upon binding to cGAMP, a catalyzed dsDNA product of cytosolic GMP/AMP synthase (cGAS) (55). Activation of STING leads to secondary activation of transcription factor IRF3 by facilitating IRF3 interaction with Tank Binding Kinase 1 (TBK1), phosphorylation of IRF3 (pIRF3), pIRF3 dimerization and translocation into the nucleus where it transactivates IFNβ and other pro-inflammatory genes (55).

As a consequence of defects in the expression/functionality of DNA repair proteins, tumors are commonly characterized by genetic instability (56, 57) and contain high concentrations of cytoplasmic DNA leading to intrinsic cGAS/STING activation and secretion of proinflammatory mediators (58–60). Progressively growing tumors have been reported to develop defects in the STING signaling pathway to avoid STING-induced apoptosis and immune surveillance (60–62). Nevertheless, dying tumor cells still release dsDNA (and/or 2’3’ cGAMP, its cGAS catalyzed product) into the TME, which may result in the activation of STING^+^ cells in the tumor stroma, including DC and VEC (63–65). This intrinsic inflammatory process may be therapeutically enhanced by local or systemic delivery of synthetic STING agonists (66, 67).

**Figure 1:**
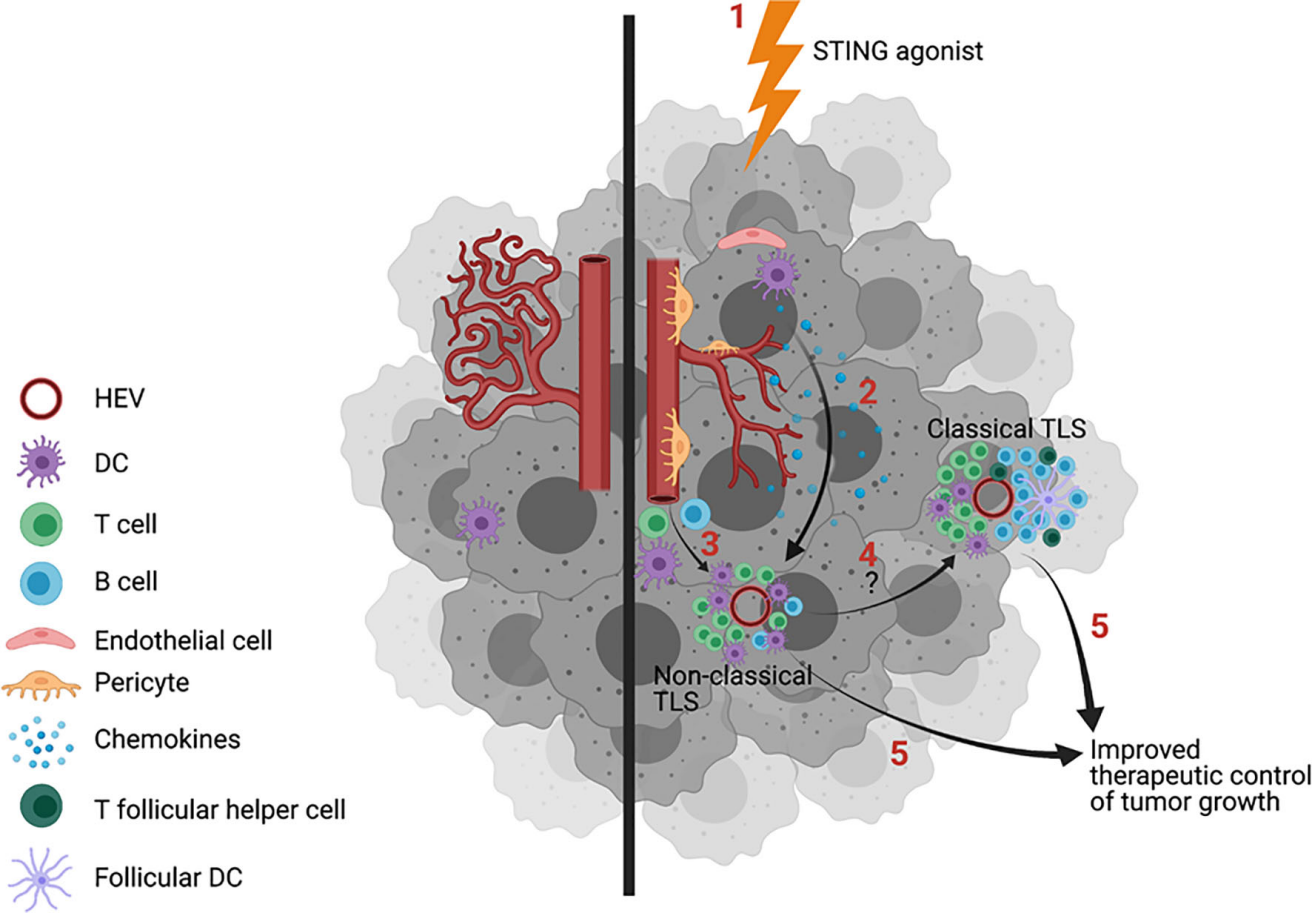
Treatment of tumors with STING agonists induces vascular normalization (VN), increased inflammatory immune cell infiltration and TLS formation. Untreated tumors exhibit dysfunctional blood vessels that limit immune cell entry into the tumor microenvironment (TME) in support of tumor progression (left). Provision of STING agonists (1, right) into the TME leads to the activation of STING^+^ stromal cells (2), including dendritic cells (DC) and vascular endothelial cells (VEC), leading to enhanced endothelial cell expression of adhesion molecules, improved vascular integrity/perfusion, and pro-inflammatory immune cell infiltration. A subset of therapeutically-conditioned VEC may differentiate into PNAd^+^ high endothelial venules (HEV). STING activated DC and HEV produce TLS-promoting cytokines/chemokines CCL19, CCL21, lymphotoxins, CXCL10 and IFNβ, which serve to recruit T cells and DC into the TME in support of non-classic, immature TLS formation proximal to HEV (3). CXCL13, required for optimal B cell recruitment into the TME and for germinal center (GC) formation within TLS, is only poorly produced in the STING agonist-conditioned tumors, precluding formation of classical, mature TLS. Combination protocols will likely be required for conversion of immature TLS into B cell/GC-rich classical, mature TLS (4) and/or to improve the therapeutic benefits associated with treatment-induced TLS formation (5). Image created with BioRender.com.

Activation of STING in tumor-associated VEC leads to VN (67, 68) characterized by increased vascular perfusion and upregulated expression of E-selectin/CD62E, VCAM-1 and ICAM-1 which facilitates circulating immune cell adhesion to the endothelium and consequent recruitment of tumor-infiltrating lymphocytes into the TME (63, 67, 68). This operating paradigm may underlie observations of cancers with reduced DNA repair proficiency and high comparative mutational burden presenting with brisk proinflammatory immune cell infiltrates (i.e. “hot tumors”) that are more prone to develop TLS (69, 70) and to be more responsive to interventional immunotherapy (71, 72). Notably, provision of low doses of STING agonists cGAMP and ADU-S100 (aka ML-RR-S2-CDA, MIW815) coordinately promote VN and CD8^+^ T cell-dependent control of tumor growth in murine models of breast carcinoma, lung carcinoma and melanoma (67, 68, 73). Yang et al. (68) further confirmed the importance of STING agonist-induced Type-I IFN produced by tumor VEC with the therapeutic benefits associated with this treatment approach. Most recently (**Figure 1**), Chelvanambi et al. has demonstrated that VN induced by intratumoral administration of low doses of the STING agonist ADU-S100 results in sustained inflammation within the TME of B16 melanomas and local production of homeostatic cytokines/chemokines (LTα, LTβ, LIGHT, CCL19 and CCL21, but remarkably not CXCL13) and pro-inflammatory/pro-TLS mediators (CXCL10, IL-36β, IFNβ) (67). These therapy-associated changes were associated with coordinate neogenesis of non-classical TLS and the development of a unique tumor-infiltrating T cell receptor (TCR) repertoire in the TLS^+^ TME that was not detectable in the peripheral immune cell compartment (67). Parallel studies using STING-KO mice confirmed the strict requirement for STING expression in host but not tumor cells for therapeutic response to intratumoral administration of ADU-S100, including TLS formation and slowed tumor growth (67).

## Combination STING Agonist Therapies May Be Required to Promote the Neogenesis of Classical, Mature TLS in the TME

The finding that STING agonists promote the formation of non-classical/immature TLS is consistent with the inability of these agents to augment production of CXCL13 within the treated TME, a prerequisite for CXCR5^+^ B cell/Tfh cell recruitment and the formation of GC within classical/mature TLS (67). While the exact mechanism underlying this deficiency in CXCL13 production remains unclear, it could relate to the known regulatory action of STING signaling in B cells. For instance, the activation of STING in B cells *via* genetic engineering to express a constitutively activated form of STING or by treatment of B cells with STING agonists results in endoplasmic reticulum (ER)-associated degradation of membrane-bound immunoglobulin, muted BCR signaling *via* enhanced SHP1 phosphatase activity and increased rates of B cell apoptosis (74–76).

Since STING agonist-associated treatment benefits occur in association with non-classical/immature TLS formation, with seemingly minimal input from B cells, these findings also reenergize discussions related to the operational importance of B cells in therapeutic anti-tumor immune responses. Despite several recent reports citing the association of B cells and GC within tumor-associated TLS and positive clinical prognosis and response to interventional immunotherapy (19, 77–80), the literature is balanced by observations for an immunosuppressive influence for intratumoral B cells in promoting tumor progression, poor patient prognosis and immune-related adverse events (irAEs) in response to immunotherapy (80–82). Translational modeling in murine tumor models has similarly provided equivocal findings. Hence, B cell deficiency (muMT) or B cell depletion (using anti-CD20 mAbs) has resulted in either decreased (83–86) or increased (87) tumor growth. In the former cases, B cells are hypothesized to serve as intrinsic immunoregulatory cells or facilitators of Treg recruitment/development/function (82–85), while in the latter situation, B cells are believed to serve as supportive antigen-presenting cells and/or producers of pro-TLS cytokines (i.e. LIGHT) and therapeutic anti-tumor antibodies (19, 78, 88–93).

In recent years, more attention has been devoted to discerning the impact of B cells in TLS that form in patients’ tumors with a generally beneficial role for B cells emerging. In both melanoma and renal cell carcinoma, B cell gene signatures are enriched in the tumors of patients who respond to immune checkpoint blockade with positive correlations observed at baseline and on-treatment (79). When tumor samples were histologically analyzed, TLS containing B cells were more commonly identified in tumor biopsies obtained from clinical responders vs. non-responders, and these mature TLS appeared more secondary-follicle-like and contained CD21^+^ follicular DC and CD23^+^ germinal center B cells (79). Furthermore, the presence of B cells in classical, mature TLS was associated with T cells exhibiting more activated, functional phenotypes and expanded repertoires (79, 94–96).

If these more recent observations can be generalized, they suggest that optimal benefits from interventional immunotherapies may require treatment-associated development of classic, mature TLS containing B cells. As such, STING agonist-based regimens should be combined (synchronously or potentially after STING agonists) with co-treatments that coordinately induce the entry of therapeutic B cells, as well as FDC and Tfh cells, into the TME to improve TLS-associated anti-tumor immune responses. Candidate co-therapies include a range of toll-receptor agonists (97–104), agonist anti-TNFR1 antibodies (105) and DNA methylase inhibitors (106) which have each been reported to augment production of CXCL13 by stromal cell populations. This augmentation would be expected to improve tumor infiltrating B cell content and GC formation within the TLS^+^ TME, conceivably improving immune-mediated control of tumor growth. Indeed, several recent reports support therapeutic synergy using treatment regimens combining STING agonists and TLR1/2 agonist Pam3Csk (97), TLR4 agonist monophosphoryl lipid A (102), TLR7/8 agonist MEDI9197 (103) or TLR9 agonist CpG (104). Although the impact of these interventional protocols on TLS formation within the TME and the evolving anti-tumor immune response remains unknown, these aspects are expected to be actively pursued in future studies.

## Combining STING Agonists With Agents Capable of Antagonizing Compensatory Regulatory Pathways for Improved Therapeutic Efficacy

Given the ability of STING agonists to promote robust pro-inflammatory responses in tumor-associated stromal cells, it is perhaps not surprising that these agents are competent to initiate the development of non-classical, immature TLS within the TME (67). And even though treatment of tumor-bearing mice with STING agonists leads to reduced levels of tumor-associated myeloid-derived suppressor cells (MDSC) and Treg cells (107, 108), these regimens promote compensatory activation of immune regulatory pathways by augmenting expression of arginase-2 (ARG2), cyclooxygenase-2 (COX2/PTGS2), indoleamine 2,3 dioxygenase (IDO), programmed death-1 (PD-1), programmed death ligand-1 (PD-L1) and prostaglandin E synthase (PTGES) within the TME (67, 109–111). Hence, combined treatment protocols that include STING agonists and antagonists of these regulatory pathways would be anticipated to enhance/sustain inflammation within the TME in support of TLS formation/maintenance and improved host control of tumor growth. While the formation of TLS has yet to be investigated as a therapeutic endpoint in translational models of such combination treatment protocols, therapeutic synergy has been observed for regimens combining STING agonists with inhibitors of COX2 (Celecoxib) or IDO (BMS-986205), or antagonist anti-PD1 and/or anti-PD-L1 antibodies (107, 109, 112).

## Discussion and Future Perspectives

TLS are increasingly viewed as important operational components supporting the development and maintenance of protective immune responses that impact patient prognosis and response to interventional immunotherapy. The ability to predictably and reproducibly promote or augment TLS formation in a patient’s tumor(s) *via* the administration of therapeutic agents may dramatically improve objective response rates over those currently observed for standard of care treatments, including immune checkpoint blockade antibodies. Although previous animal modeling of gene therapy and targeted antibody approaches to deliver individual TLS homeostatic cytokines/chemokines have proven successful in controlling tumor growth and promoting TLS formation in mice (113–115), these strategies have yet to be effectively translated into the clinic, and they rely on the biologic dominance of a single agent to initiate the complex biologic process of TLS formation. In this regard, STING agonism provides the opportunity to coordinately activate a range of tumor-associated stromal cell populations, including vascular endothelial cells and immune cells, leading to VN, enhanced immune cell infiltration, and the establishment of a pro-inflammatory TME in which TLS-associated homeostatic chemokines and cytokines are produced and TLS formation is facilitated. While provision of STING agonist ADU-S100 into B16 melanomas resulted in the development of a T cell repertoire unique to the therapeutic TLS^+^ TME and to some abscopal benefit in regulating the growth of distal, untreated tumor lesions, the current approach has several limitations.

First, the approach involves direct injection of a second-generation STING agonist (which can only be administered locally) into an accessible lesion, with the intent to treat disseminated disease. In this regard, while local injection of STING agonists [i.e. ADU-S100/MIW815 (NCT02675439), MK-1454 (NCT03010176)] as monotherapies has provided some evidence for pro-inflammatory changes in the TME or patient sera, therapeutic benefits have been minimal (i.e. < 5% objective response rate) in early phase clinical trials treating advanced-stage cancer patients (as described in greater detail in a series of recent outstanding reviews) (55, 116, 117). This deficiency may be circumvented by the provision of next-generation, systemic STING agonists for more effective treatment of patients with multifocal, disseminated disease in visceral tissue sites. Several of these agents (i.e. E7766 [NCT04109092], GSK-3745417 [NCT03843359], MSA-2, SB-11285 [NCT04096638], TAK-676 [NCT04420884]) (55, 118, 119) are planned for, or are currently being evaluated in, phase I clinical trials. Given the pro-apoptotic impact of high-doses of STING agonists on VEC (i.e. vasoablative) and immune cell populations (67, 73–76, 120, 121), but the ability of low-dose regimens to promote VN and enhanced pro-inflammatory immune function in pre-clinical models, it might be anticipated that low-dose protocols will provide optimal immunotherapeutic benefits in these trials. While it is not clear that the formation of TLS represents an exploratory endpoint in these ongoing trial designs, one might expect that low-dose regimens of these next-generation STING agonists will prove effective in inducing *de novo* development of TLS or expansion of existing TLS within the tumor of treated patients. It is also possible given enhanced autoimmune manifestations in older (cancer) patients (122), many of which have known associations with the formation of TLS in affected tissues (123), that treatment with systemic STING agonists may exacerbate the incidence and severity of irAEs.

Second, the TLS promoted by ADU-S100 in the TME appear rich in CD8^+^ T cells, DC and HEV, but they are poor in CXCL13 production and infiltrating B cell/GC content (67) [i.e. representative of non-classical, immature TLS (2, 16)]. If B cells are indeed crucial to superior therapeutic benefits associated with TLS formation in tumors, additional co-therapies that i.) reinforce local CXCL13 production, ii.) B cell, Tfh and FDC infiltration and iii.) GC formation, may need to be combined with STING agonists to achieve maximal interventional benefit.

Third, the natural checks and balances in evolving immune responses must be considered in conditionally optimizing STING agonist-based immunotherapies. The robust pro-inflammatory responses evoked by these agents result in an upregulation in immune regulatory pathways within the TME, including but not limited to prostaglandin E production and immune suppression mediated by arginase, IDO and co-inhibitory receptors (67, 68). These regulatory pathways may be antagonized (individually or collectively) in combination STING agonist protocols using available, in-clinic targeted inhibitors. Such approaches would be expected to augment and prolong inflammation within the TME in support of TLS formation and the mobilization of broadly-reactive anti-tumor immune responses in the therapy setting. However, as suggested above, such deregulated reinforcement of TLS formation in tumors and normal tissues carries increased risk for the evolution of severe (autoimmune) irAEs.

Finally, previous findings suggest that in certain cases, STING activation and the presence of HEV/TLS may be associated with tumor progression. Hence, in murine lung carcinoma models (109, 111), provision of STING agonists (i.e. CDA) initially slowed primary tumor growth but ultimately resulted in disease progression and metastasis due to treatment-associated enhancement in immune regulatory/tolerogenic pathways (COX2, IDO, PD-1), which could be mitigated using targeted inhibitors in combination protocols (109, 111). Furthermore, tumors with pronounced chromosomal instability and intrinsic STING signaling competency have been reported to exhibit STING-dependent metastatic potential (124), which might be envisioned to be further exacerbated by treatment with STING agonists. Additional reports caution that TLS enriched in Treg cells or immature (thin-walled) HEV may be associated with poor immune infiltration of tumors, poor patient prognosis and increased tumor metastasis (125). Therefore, baseline tumor STING signaling competency and the quality and cell composition of STING-agonized TLS should be carefully monitored for correlative impact on cancer patient outcome.

## Author Contributions

All authors contributed to the article and approved the submitted version.

## Funding

This work was supported by NIH grants R01 CA204419 and P01 CA234212 (both to WS) and NIH T32 CA082084 (to JF).

## Conflict of Interest

The authors declare that the research was conducted in the absence of any commercial or financial relationships that could be construed as a potential conflict of interest.
